# Green Diesel Production by Catalytic Hydrodeoxygenation of Vegetables Oils

**DOI:** 10.3390/ijerph182413041

**Published:** 2021-12-10

**Authors:** Giuseppe Di Vito Nolfi, Katia Gallucci, Leucio Rossi

**Affiliations:** 1 Dipartimento di Scienze Fisiche e Chimiche, Università degli Studi dell’Aquila, 67100 L’Aquila, Italy; giuseppe.divitonolfi@graduate.univaq.it; 2 Dipartimento di Ingegneria Industriale e dell’Informazione e di Economia, Università degli Studi dell’Aquila, 67100 L’Aquila, Italy; katia.gallucci@univaq.it

**Keywords:** hydrodeoxygenation reaction, catalysis, green diesel, renewable raw materials

## Abstract

Non-renewable fossil fuels and the air pollution associated with their combustion have made it necessary to develop fuels that are environmentally friendly and produced from renewable sources. In addition, global warming and climate change have brought to the attention of many countries the need to develop programs and reforms, such as the 2030 Agenda of the United Nations and the European Green Deal, that finance and promote the conversion of all socio-economic activities in favor of sustainable and environmentally friendly development. These major projects include the development of non-polluting biofuels derived from renewable sources. Vegetable oils are a renewable source widely used to produce biofuels due to their high energy density and similar chemical composition to petroleum derivatives, making them the perfect feedstock for biofuel production. Green diesel and other hydrocarbon biofuels, obtained by the catalytic deoxygenation of vegetable oils, represent a sustainable alternative to mineral diesel, as they have physico-chemical properties similar to derived oil fuels. The catalyst, temperature, hydrogen pressure, and the type of vegetable oil can influence the type of biofuel obtained and its properties. The main aspects discussed in this review include the influence of the catalyst and reaction conditions on the catalytic deoxygenation reaction.

## 1. Introduction

The growing interest in the development of alternative energy sources to fossil fuels is linked to the known problems deriving from their use such as the environmental impact and the depletion of reserves. Unstoppable technological development has as an inevitable consequence a progressive increase in world energy demand. The World Oil Outlook 2019 reports the increase in energy demand from 2018 to 2040 (Table 1), distinguishing between OECD (Organization for Economic Co-operation and Development) and non-OECD countries, foreseeing a significant increase in global energy demand, mainly caused by developing countries [1].

**Figure 1:**
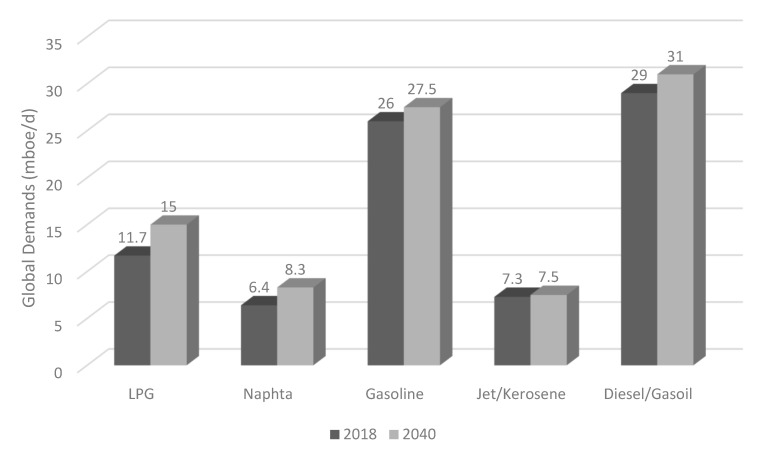
Increase in energy demand for oil derivatives between 2012 and 2035.

**Figure 2:**
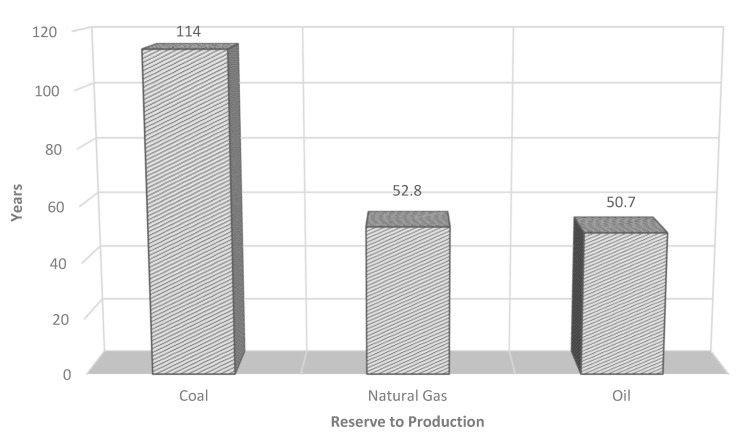
Estimated time for fossil fuel depletion [2].

An increase of about 71 million barrels of oil per day over 22 years is expected, and this increase is given exclusively by non-OECD countries. Moreover, it is estimated that oil will dominate the energy sector until 2040, contributing to global energy needs of 31% in 2018 and 28% in 2040. In addition, it is expected that the demand for petroleum derivatives will continue to grow (Figure 1). Therefore, the progressive decrease in fossil fuel reserves and mainly oil reserves is inevitable. Assessing the reserves-to-production (R/P) ratio (Figure 2), it is estimated that oil reserves will be depleted in 50 years (note that these data refer to 2015 and are subject to continuous change due to the discovery of new reserves and the annual increase in consumption) [2].

Fossil fuel consumption also has a severe environmental impact. Many pollutant compounds such as greenhouse gases (CO_2_), nitrogen and sulfur oxides (responsible for acid rain), volatile organic compounds (VOCs), and particulate matter are generated during the combustion of fossil fuel derivatives [3,4]. As a result, there is a growing interest in the development of renewable and non-polluting energy. In fact, on 25 September 2015, the United Nations (UN) approved “Transforming our World: the 2030 Agenda for Sustainable Development”, which is a project aimed to achieve global sustainable development ensuring that “no one is left behind”. To reach the goal of this project, it is important that every aspect of sustainable development, economic, social, and environmental, are interconnected [5]. In addition to this project, the European community has undertaken the project of the European Green Deal, which provides a growth strategy in order to make the European Union an organization with a modern and competitive economy aimed at becoming as efficient as possible from the point of view of resources and, in particular, achieving zero net emissions of greenhouse gases by 2050 [6]. The Green Deal agrees with the 2015 Paris Agreement that foresees the reduction of greenhouse gas emissions with the aim of reducing global warming by 1.5 °C compared to pre-industrial levels [7]. From what has been said, it is clear that the development of biofuels is a very important subject with positive environmental implications too.

**Figure 3:**
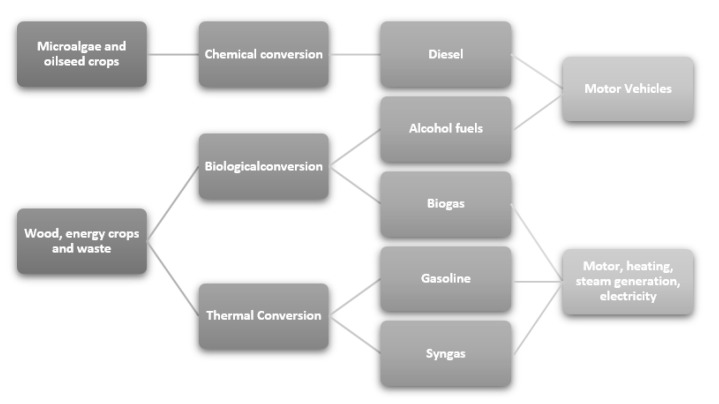
Schematic representation of processes to produce biofuels (Figure modified from [4]).

Solar, wind, and geothermal energy represent some examples of renewable energies. Alternatively, the energy obtained by biomasses is the main renewable energy source for the production of solid, liquid, and gaseous biofuels [8]. Biomass is defined as any material of biological origin derived from wood, agricultural crops, livestock, marine waste, and also man-made waste [9]. The use of biomass for energy production can indeed bring enormous benefits from an environmental and economic point of view. Moreover, being a renewable resource, biomass is always available [10]. Biomass can be roughly divided into three main classes: carbohydrates, triglycerides, and waste biomass. Biofuels are defined as the fuels, solid, liquid, and gaseous, that are environmentally friendly, biodegradable, and non-toxic and obtained by the treatment of biomass. They can represent an effective and sustainable alternative to conventional fossil fuels [11]. Beyond the obvious advantage of renewability, the development of biofuels from plant biomass can significantly reduce greenhouse gas emissions. In fact, the CO_2_ emission by their combustion is part of a CO_2_ cycle in which the CO_2_ emitted is reused by plants during their life [10]. In addition, especially in the case of biofuels derived from vegetable oils, there is a significant decrease in the emission of sulfur oxides, as plants are very poor in sulfur [12]. Biofuels can be produced by biochemical processes (alcoholic fermentation of sugars or anaerobic digestion of biomass) or by thermo-chemical processes (catalytic deoxygenation, pyrolysis, thermal, and/or catalytic cracking of biomass) (Figure 3) [4]. As reported by Saladini et al., the proper classification of biofuels depends on numerous factors, but for simplicity, they can also be classified according to the feedstock used for their production [13,14]. First-generation biofuels are typically produced from edible oil-based plant feedstock. Second-generation biofuels derive from non-food crops and therefore do not compete with the food industry (as in the case of first-generation biofuels). Third-generation biofuels are produced from aquatic cultivated feedstock (algae), and finally, fourth-generation biofuels are obtained from genetically modified algae to improve the production of biofuel [14]. Examples of first-generation biofuels are bioethanol, biogas, and biodiesel (fatty acid methyl esters, FAME), while second-generation biofuels obtained from edible and non-edible feedstock are hydrocarbon fuels obtained by catalytic deoxygenation or the Fischer–Tropsch process [15].

Vegetable oils are suitable substrates for hydrocarbon biofuel production as they have, similar to petroleum derivatives, high energy density and a simple chemical composition [16]. Vegetable oils can be directly used in diesel engines, but high viscosity and low volatility may cause engine problems [17,18]. On the other hand, the appropriate treatment of vegetable oils allows the production of hydrocarbon biofuels that are fully compatible with fossil fuel derivatives. Biodiesel, produced by transesterification of vegetable oils, and green diesel (or renewable diesel), obtained by the catalytic deoxygenation of vegetable oils, are alternative fuels to mineral diesel.

## 2. Green Diesel vs. Biodiesel

**Figure 4:**
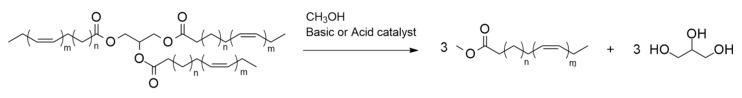
Transesterification reaction for biodiesel production.

Biodiesel is the product obtained from the transesterification of edible and non-edible vegetable oils with methanol or ethanol in the presence of acid or basic catalysts [19]. The fatty acids contained in vegetable oil triglycerides are converted into the corresponding methyl esters (FAME) by the transesterification reaction (Figure 4).

The main advantages of biodiesel relay to the use of renewable materials and to the reduction of polluting gas emissions. Moreover, the production process leads to high yields and requires mild operating conditions [20]. However, several drawbacks, such as high oxygen content, high corrosivity, storage instability, and limited miscibility with conventional fossil fuels, make biodiesel an unsuitable substitute for petrol diesel [21]. A valuable alternative to biodiesel is green diesel, which is obtained by the catalytic deoxygenation (DO) of vegetable oils. DO is a thermal process, typically conducted in a hydrogen atmosphere and with a heterogeneous metal catalyst, in which vegetable oils are converted into hydrocarbons. A DO commercial process was patented by Neste Oil [22]. This treatment produces a biofuel mainly consisting of *n*-alkanes, which is therefore chemically similar to mineral diesel, making it completely compatible and miscible in every proportion. The differences between biodiesel, green diesel, and petrol diesel can be seen in Table 2 [23].

Both green diesel and biodiesel have a higher cetane number than conventional petrol diesel (higher for green diesel). Biodiesel is chemically different from petrol diesel. In fact, it has a high oxygen content and as a result a lower calorific value, energy density, and energy content [24]. In contrast, green diesel is chemically similar to petrol diesel and more suitable than biodiesel as its substitute. Both biodiesel and green diesel have low sulfur emissions [12], but unlike green diesel, biodiesel has higher nitrogen emissions. CO_2_ emissions are also significantly reduced. Han et al. [25] observed a reduction of greenhouse gas emissions of 41–63% in the case of jet biofuels produced by catalytic deoxygenation. Compared to green diesel, biodiesel has better properties as a lubricant, and it also has a higher flash point. In contrast, it has poorer oxidation stability, storage, and also poor cold flow properties [26]. On the other hand, green diesel has high oxidation stability, storage stability, and when properly treated by a hydroisomerization reaction (a reaction in which the *n*-alkanes of green diesel are partially converted into the corresponding branched isomers) it also has excellent cold properties [27]. Furthermore, the different chemical composition between biodiesel and petrol diesel implies that the former can only be used in a mixture with conventional diesel. In fact, it can be used pure only after the adaptation of the engine [26]. Green diesel, on the other hand, being chemically similar to petrol diesel, can completely replace conventional diesel.

## 3. Catalytic Deoxygenation (DO) Process

**Figure 5:**
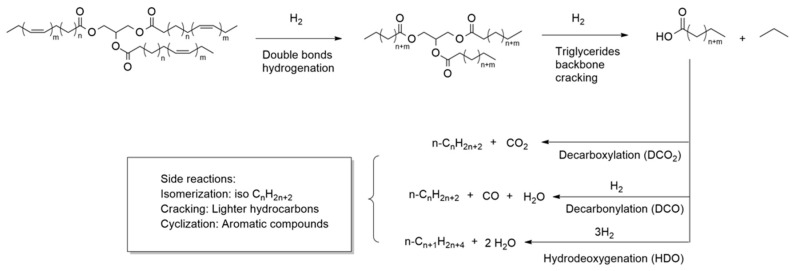
Possible reaction pathway during catalytic deoxygenation of vegetable oil (figure modified from [29]).

Catalytic deoxygenation (DO) is a thermic treatment, in a hydrogen atmosphere and with a heterogeneous catalyst, in which the triglycerides of vegetable oils are converted into hydrocarbon compounds. Gosselink and co-workers have written an exhaustive review on the reaction pathways that occur during DO over a model compound or real feedstock [28] (Figure 5).

**Figure 6:**
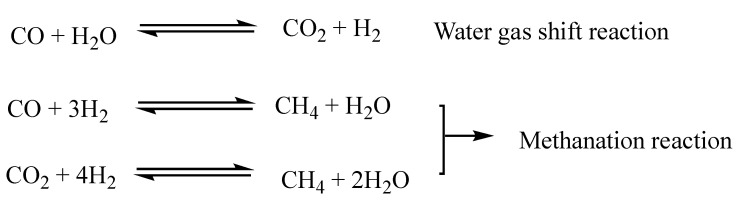
Gas phase reactions in catalytic deoxygenation of vegetable oils.

As suggested by Kubicka et al., the first step of DO involves the hydrogenation of the double bonds of unsaturated fatty acids [30]. Then, the triglycerides are broken down to obtain free fatty acids. The most common mechanism of triglyceride cleavage is a β-elimination reaction in which the triglyceride is converted into a free fatty acid and an unsaturated diglyceride. The diglyceride needs to be re-hydrogenated before undergoing further β-elimination to release a second fatty acid molecule. The third step of hydrogenation and β-elimination leads to the release of the third fatty acid and a propane molecule [31]. The free fatty acids can then follow three different reaction pathways according to the selectivity of the process: (a) a hydrodeoxygenation reaction (HDO), which forms an alkane with the same number of carbon atoms as the starting fatty acid and water; (b) decarbonylation (DCO); (c) decarboxylation (DCO_2_) reactions in which an alkane with a carbon atom less than the starting fatty acid is formed and the oxygen is released as CO and H_2_O (DCO) or CO_2_ (DCO_2_) [32]. Reaction selectivity can be driven by varying operating parameters, such as temperature and pressure, but also by varying the nature of the metal catalysts and the supporting material. The HDO route is preferable from the point of view of atom economy because it produces H_2_O as a by-product, though it consumes more hydrogen. In addition, water produced during the reaction could deactivate the catalyst [33]. The DCO_2_ route does not consume hydrogen but, on the other hand, it has a lower atom economy and produces CO_2_, which is a greenhouse gas. The catalytic deoxygenation produces a liquid product consisting of *n*-alkanes. Since it is chemically similar, green diesel is fully compatible with conventional diesel, and they can be mixed in any proportion. At this stage, however, the produced biofuel is not very suitable because it has poor cold properties [34]. Through the hydroisomerization process, a DO-similar process, the linear *n*-alkanes are converted into the corresponding branched isomers leading to a product with better cold properties [35]. The process is generally conducted at higher temperatures than DO; zeolite-supported metals (such as Pt/SAPO-11 where SAPO-11 are a medium-pore silicoaluminophosphate molecular sieve) are used as catalysts. Interestingly, after the isomerization reaction, the biofuel retains a similar cetane value [36]. Cracking and other secondary reactions favored by high temperatures are possible in DO. In these reactions, alkanes and fatty acids undergo a thermal cleavage yielding short-chain compounds. This reaction is an undesirable event if the intention is to produce a green diesel type of biofuel, while it is desirable for producing jet fuels or gasoline range biofuels [37]. Another possible side reaction is the aromatization reaction (cyclization of saturated alkanes followed by dehydrogenation). Normally, it is an undesirable reaction, as the aromatic compounds lower the calorific value of the fuel and lead to the deactivation of the catalysts. Nevertheless, in the case of jet fuels, a small percentage of aromatics is desirable [37]. Another possible reaction is the coking reaction in which alkanes, or aromatic compounds, can polymerize to form a carbonaceous residue consisting of heavy hydrocarbons. This reaction is highly undesirable because the presence of coke strongly deactivates the catalyst by adsorbing on it and blocking its pores. It is possible to reduce the effect of secondary reactions by changing the reaction parameters. The reactions mentioned so far take place in the liquid phase, but gas phase reactions are also possible (Figure 6).

Considering the water–gas shift reaction, it is evident that driving the reaction towards H_2_ production would be profitable from the point of view of H_2_ consumption, but at the same time, it would be necessary to counteract methanation reactions that consume large quantities of hydrogen. DCO/DCO_2_ consume less H_2_ than HDO, but the formation of CO and CO_2_ in addition to strong methanation could lead DCO/DCO_2_ reactions to consume more H_2_ than HDO [38].

Catalytic deoxygenation can be affected by a large number of variables: the nature of the catalyst, temperature, reaction atmosphere, hydrogen pressure, solvent (whatever the reaction that was conducted in the presence of a solvent), the substrate, and also the reactor used. All these parameters influence conversion, reaction selectivity, hydrocarbon mixture distribution (gasoline range, jet fuel range, diesel range), isomerization, coke formation, and catalyst deactivation.

### 3.1. Catalyst and Support

As previously reported, DO reaction is a thermic, energy demanding treatment. The use of a catalyst reduces this demand. Here, we report the more promising catalysts for energy-saving and cost-effective processes, thanks to milder operating conditions. The choice of the active phase and support can greatly influence the distribution of products in the liquid phase and the conversion of the feedstock. The most commonly used catalysts are sulfided transition metal catalysts such as Mo and W doped with promoters such as Ni and Co (HDS catalysts). Kubicka et al., investigating the DO of rapeseed oil over sulfided NiMo/γ-Al_2_O_3_, Ni/γ-Al_2_O_3_ and Co/γ-Al_2_O_3_, observed that the bimetallic catalyst was more active than the monometallic ones. Moreover, Ni is more selective towards DCO/DCO_2_ and Mo towards HDO. The same amounts of the products deriving from both reactions were obtained using NiMo [39]. Comparative analysis of sulfided NiMo, NiW, CoMo, and CoW, supported on γ-Al_2_O_3_, SiO_2_, TiO_2_, SBA-15 (Santa Barbara amorphous-15 mesoporous silica), and HT (hydrotalcites layered double hydroxides), in rapeseed oil hydrotreatment, was performed by Horceck et al. [40]. They concluded that the most active catalyst was NiMo/γ-Al_2_O_3_. NiMo/γ-Al_2_O_3_ was also more active than NiCoMo and NiCoW trimetallic catalysts. They also studied the effect of the support, observing that the most active support was alumina together with SBA-15. If compared to SiO_2_, SBA-15 shows greater activity and different selectivity; with SBA-15, there is a greater prevalence of HDO, while SiO_2_ prefers DCO/DCO_2_. This is due to the high surface area of SBA-15 (650 m^2^/g) compared to SiO_2_ (57 m^2^/g) that improves the diffusion of the reagents inhibiting the breaking of the C–C bonds (and thus the DCO/DCO_2_) [40]. These catalysts are very active but also have the significant disadvantage of rapid deactivation via sulfur leaching. A catalyst’s deactivation by leaching can be minimized by using a sulfiding agent that reduces the leaching of the catalyst [41]. Senol et al. have also analyzed the effect of two sulfiding agents, H_2_S and CS_2_, observing that H_2_S was more effective than CS_2_. They actively participate in promoting DO by increasing the acidity of the catalyst and preventing catalyst deactivation. However, this leads to the formation of pollutant gases and contamination of the biofuel with sulfur [42]. Because of these limitations, the scientific community has focused on the development of non-sulfided catalysts. Generally, these catalysts are based on noble metals because are they are generally more active, although more expensive, than the corresponding reduced-transition metals [32,43]. Snare et al. compared the activity of several noble metals (Pd, Pt, Ir, Ru, Os) and Ni supported on AC (activated carbon), γ-Al_2_O_3_, Cr_2_O_3_, and SiO_2_ in the DO of oleic acid. It was evident that the most active catalysts were based on Pd and Pt, followed by Ni [32]. Morgan et al., investigating the hydrotreatment of tristearin, triolein, and soybean oil over 20 wt% Ni/C, 5 wt% Pd/C, and 1 wt% Pt/C under nitrogen atmosphere, observed that a sufficiently high content of supported metal can lead a Ni-based metal catalyst to be more active than a noble metal catalyst [44]. Additionally, Veriansyah et al., comparing the activity of monometallic Pd, Pt, and Ni-based catalysts with bimetallic catalysts such as reduced NiMo in the DO of soybean oil, observed that NiMo is also more active than noble catalysts [29]. Metal transition-based catalysts can also be a good alternative to typical sulfided catalysts. In fact, by comparing the activity of reduced bimetallic catalysts with sulfided bimetallic catalysts, Harnos et al. suggest that non-sulfided catalysts may be a valuable alternative due to their comparable activity and higher stability than the corresponding sulfided catalyst [45]. As an alternative to the above-mentioned catalysts, phosphide, carbide, and nitride catalysts have also been developed [34]. Table 3 resumes the results obtained in the DO process of several triglycerides and model compounds using different metal catalysts.

In all cases reported, it is evident that the choice of the active phase is as much important as the choice of the support. Snare et al. have correlated the increased activity of their catalyst supported on activated carbon with its high surface area, leading to lower deactivation via sintering and coking [32]. The beneficial effect of a support with high superficial area was also reported by Wang et al. in the hydrotreatment of soybean oil over NiMo carbide catalysts. They observed that the best conversion (100%) and diesel selectivity (97%) were achieved with the lab-made NiMo/Al-SBA-15 (zeolite SBA-15 enriched with Al), which has the highest surface area and the largest porosity [46]. The correlation between support surface area and catalyst activity has been observed by several authors [47,48,49,50]. The acidity of the support is another parameter that can affect the deoxygenation reaction. Peng et al., in the DO of palmitic acid over Pd, Pt, and Ni supported on ZrO_2_, Al_2_O_3_, HZSM-5 (hydrogen form of zeolite Socony Mobil-5), HBEA (hydrogen form of β-zeolite) and C, reported that metal supported on a support with weak or medium acidity, such as ZrO_2_ and zeolites, showed increased catalytic activity [51]. Furthermore, in another work, they correlate the increased activity of catalysts supported on ZrO_2_ with its reducible oxide properties which, through oxygen vacancies, actively participate in the reaction by adsorbing oxygenated compounds [52].

**Table 3:** Catalytic deoxygenation of vegetable oils and related compounds with different catalysts.

Type	Catalyst	Support	Feedstock	Reaction Condition			Conversion (%)	Selectivity (%)	Ref.
				T (°C)	P (Bar)	T (h)			
Sulfided	NiMo, CoMo, NiW	Al_2_O_3_, B_2_O_3_-Al_2_O_3_	Waste cooking oil	250–350	70	3	100	87% *n*-C_15_-C_18_ 7.5% *i*-C ^1^	[53]
	NiMo	SiO_2_, Al_2_O_3_, HY ^2^, HZSM-5 ^3^, SiO_2_-Al_2_O_3_	Jatropha oil, palm oil, canola oil	350	40	LHSV ^4^ = 7.6 h^−1^	100	90% *n*-C_11_-C_20_	[54]
	NiW NiMo	SiO_2_-Al_2_O_3_ Al_2_O_3_	Waste soybean oil, refinery oil	340–380	50	LHSV = 2.4 h^−1^	>99	96–83% *n*-C_15_-C_18_ (NiMo) 79–43% *n*-C_15_-C_18_ (NiW)	[55]
	NiMo NiW	Al_2_O_3_ TiO_2_, ZrO_2_	Rapeseed oil, sunflower oil, palm oil, tall oil + atmospheric gas oil	320–360	20–110	LHSV = 1.0 h^−1^	100	2–4% *i*-C_15_-C_18_ 84–91% *n*-C_15_-C_18_	[56]
	NiMo	Al_2_O_3_	Palm oil	270–420	15–80	LHSV = 0.25–5 h^−1^	100	96% *n*-C_15_-C_18_	[57]
Reduced	Ni	SiO_2_; Al_2_O_3_; SAPO-11 ^5^; HZSM-5; HY	Methyl palmitate	220	20	6	99.8	3% *i*-C_15_-C_16_ 87% *n*-C_15_-C_16_	[58]
	Ni	SiO_2_; Al_2_O_3_; HZSM-5;	Stearic acid	260–290	8	6	100	90% *n*-C_17_ 1.5% *n*-C_18_	[59]
	Ni	HPW ^6^/Al_2_O_3_	Jatropha oil	320–380	33	LHSV = 1.0 h^−1^	99.8	85.5% *n*-C_15_-C_18_	[60]
	CoMo ^7^	Al_2_O_3_	Sunflower oil	300–380	40–60	LHSV = 1.0 h^−1^	100	83–89% *n*-C_15_-C_18_	[61]
	CoMo	Al_2_O_3_	Sunflower oil	300–380	20–80	LHSV = 1.0 h^−1^	100	69.5–73% *n*-C_11_-C_19_ 25–44.5% *i*-C	[62]
Noble	Pd	Al_2_O_3_	Stearic acid	350	6–14 (H_2_ or N_2_)	3	100	9% *n*-C_18_ 91% *n*-C_17_	[63]
	Pd	SBA-15 ^8^	Stearic acid	300	17 (5%H_2_/Ar)	5	96	98% *n*-C_17_	[64]
	Ru, Pd, Pt, Rh	HZSM-5	Stearic acid, methyl stearate	160–260	30	8	90.8	77% *n*-C_17_-C_18_	[65]
	Pd	C	Palmitic acid, stearic acid	300	17 (5%H_2_/Ar)	3	98	>99% *n*-C_15_-C_17_	[66]
	Pt, Pt-Re	SiO_2_, SiO_2_-Al_2_O_3_, HZSM-5, USY ^9^, BEA ^10^, HY, H-MOR ^11^, PER ^12^, L ^13^	Jatropha oil	270	65 (91%H_2_/Ar)	12	100	95% *n*-C_10_-C_20_	[67]
Carbide, Phosphide, Nitride	W_2_C, Mo_2_C	CNF ^14^	Oleic acid	350	50	5	100	85% *n*-C_18_	[68]
	Mo_2_C	AC ^15^	Methyl palmitate	280	10	4	100	4% *n*-C_15_ 91% *n*-C_16_	[69]
	Mo_2_C	RGO ^16^	Oleic acid (OA), soybean oil (SO)	350	50	LHSV = 2 h^−1^	95 (OA), 71.8 (SO)	85% *n*-C_18_	[70]
	Mo_2_C	CNF	Methyl palmitate	260	25	3	98	91.5% *n*-C_16_	[71]
	Ni_2_P	SiO_2_	Methyl laurate	300	20	WHSV ^17^ = 5.2 h^−1^	97.2	84% *n*-C_11_ 15% *n*-C_12_	[72]
	NiP	AC	Palmitic acid	350	1 (5%H_2_/Ar)	2.5	99.4	11% *n*-C_11_-C_14_ 74% *n*-C_15_	[73]
	Mo_2_N, VN,WN	Al_2_O_3_	Oleic acid, canola oil	380–410	71.5	GHSV ^18^ = 1850 h^−1^	97	84% hydrocarbons fuel	[74]
	NiMoC, NiMoN	ZSM-5	Soybean oil	360–450	45	LHSV = 1 h^−1^	100	50 wt% hydrocarbon fuel	[75]
	MoC, MoN, MoP	Al_2_O_3_	Rapeseed oil	350–390	55	LHSV = 1–4 h^−1^	--	73–80% diesel-like fuel	[76]

^1^ *i*-C = isoalkanes; ^2^ HY = hydrogen form of zeolite Y; ^3^ HZSM-5 = hydrogen form of zeolite Socony Mobil-5; ^4^ LHSV = liquid hourly space velocity; ^5^ SAPO-11 = medium-pore silicoaluminophosphate molecular sieve; ^6^ HPW = phosphotungstic acid; ^7^ commercial; ^8^ SBA-15 = Santa Barbara amorphous-15; ^9^ USY = ultrastable zeolite Y; ^10^ BEA = beta zeolite; ^11^ MOR = hydrogen form of mordenite zeolite; ^12^ FER = ferrierite zeolite; ^13^ L = zeolite type L; ^14^ CNF = carbon nanofibers; ^15^ AC = activated carbon; ^16^ RGO = reduced graphene oxide; ^17^ WHSV = weight hourly space velocity; ^18^ GHSV = gas hourly space velocity.

**Figure 7:**
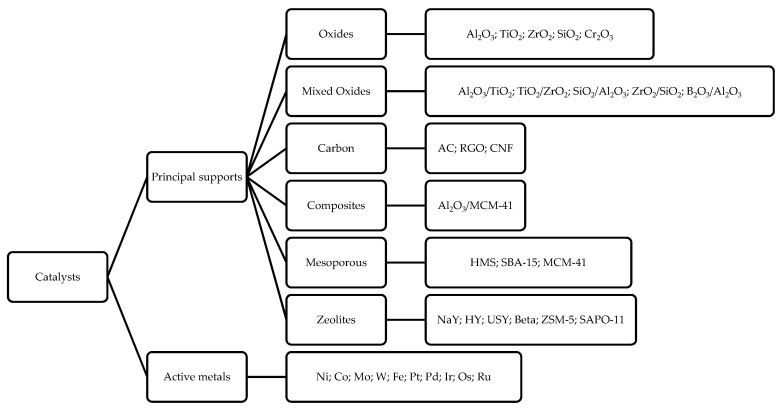
Principal metal catalysts and supports used in DO reaction.

In another work, Peng et al. analyzed the DO reaction of oil extracted from microalgae using two Ni-based catalysts supported on H-ZSM5 and H-β. With Ni/H-ZSM5 the reaction shows a high degree of cracking (43%) and coke formation [77]; the authors correlate these phenomena to the higher concentration of Bronsted acid sites of this catalyst that greatly favor cracking. They found that when increasing the zeolite’s Si/Al ratio (as the Si/Al ratio increases, the zeolite’s acidity decreases) cracking and coke formation decrease, but at the same time, the conversion decreased too. The acidity–cooking correlation has also been reported by Ardiyanti et al. in the upgrading of fast pyrolysis oil using NiCu/γ-Al_2_O_3_ and NiCu/δ-Al_2_O_3_ [78]. They observed that using δ-Al_2_O_3_ (less acidic than γ-Al_2_O_3_) leads to a minor amount of coke. Adequate acidity is necessary for triglycerides conversion, and it is necessary to avoid a too high acidity, as it favors cracking reactions and coke formation. In addition, the support can also influence the HDO, DCO/DCO_2_ reaction selectivity [51,52,77]. As observed by Twaiq et al., the size of the pores is also important. By studying the cracking reaction of palm oil over various zeolites (HZSM-5, β-Zeolite, USY), ref. [79] the authors suggest that the support pore size strongly affects the hydrocarbon distribution in the diesel mixture; USY (ultrastable Y) zeolite, which has a larger pore size, leads to less cracking (gasoline range 4–17%) than HZSM-5 zeolite (gasoline range 17–28%). The same results are obtained in aromatization, leading to a lower formation of aromatic hydrocarbon (20–38% for HZSM-5 versus 3–13% for USY). A sufficiently large pore size would tend to minimize cracking, thus leading to a greater diesel selectivity. Indeed, mesoporous materials are experiencing increasing interest as the mesoporous pores of these materials allow for easier diffusion of the substrate, which implies less coking and cracking reactions [75,79]. A scheme reassuming the principal catalysts and supports used in DO reaction is reported in Figure 7.

### 3.2. Temperature

Temperature is a very important variable in the catalytic deoxygenation reaction, as it can significantly influence rate, conversion, hydrocarbon distribution, cracking, and to a lesser extent, reaction selectivity. Snare et al., studying the DO of ethyl stearate over Pd/C, observed that an increase in temperature from 300 to 360 °C leads to a fourfold increase in conversion [80]. Similar results were reported by Madsen et al. in the oleic acid/tripalmitin mixture (1:3) hydrotreating in H_2_ atmosphere over Pt/Al_2_O_3_ [43]. The authors showed an increase in conversion from 6% at 250 °C to 100% at 325 °C. An increase in conversion with temperature has also been observed by Maki-Arvela et al., but more interestingly, they reported that an increase in temperature also leads to a higher degree of dehydrogenation as the *n*-heptadecane/*n*-heptadecene ratio decreases [81]. The effect of the reaction temperature on the dehydrogenation reaction was also observed by Cheng et al. in the hydrotreating of soybean oil over NiMo/HY (HY = hydrogen form of the zeolite Y) for the production of jet biofuel [82]. In their work, they reported an increase in the formation of aromatic hydrocarbons as the temperature rises. In fact, at temperatures above 390 °C, the aromatic content increased from 17.6 to 28.7%. A similar trend was also observed by Li et al. [83]. The temperature also has a great influence on the range of hydrocarbon distributions in the biofuel. Verma et al. found that a temperature increase (375–450 °C) leads to an increase in the distribution of hydrocarbons in the kerosene range (425 °C), indicating an improved cracking reaction with the temperature and, as consequences, they also observed an increase in isomerization selectivity as the temperature increases [84]. Working at 375 °C led to higher diesel selectivity (85–96%); if higher temperatures were used (450 °C), the cracking is prevailing, leading to a decrease in the kerosene range in favor of gaseous products. Similar results were observed by Srifa et al. [57]. The correlation between cracking and temperature has also been observed by Pinto et al. in the DO of pomace oil olives [85]. As the temperature increased, an increase in light hydrocarbons and a decrease in heavy fractions was observed. This phenomenon improves with longer reaction time. Moreover, analyzing the gas phase of the reaction, they observed that as the temperature increases, the presence of gases such as methane, ethane, and other gaseous hydrocarbons increases, indicating a greater degree of cracking. They also observed an increase in CO and CO_2_ concentration, which seems to indicate that an increase in temperature leads to higher selectivity of reaction towards DCO/DCO_2_, and this is in agreement with the endothermic nature of these reactions. On the other hand, HDO is exothermic and therefore favored at lower temperatures [86,87]. Liu et al. investigated the isomerization of palm oil over Ni/SAPO-11 (medium-pore silicoaluminophosphate molecular sieve), observing that low reaction temperatures (320 °C) yield low isomerization (in favor of a higher selectivity towards *n*-alkanes in the diesel range), while higher temperatures considerably increase isomerization activity, often accompanied by a considerable cracking. Isomerization selectivity greater than 80% and a liquid hydrocarbon yield of 70% were obtained [88]. Considering all the above reported, it is evident that temperature control is crucial in order to obtain the desired type of fuel. Moreover, the temperature also plays an important role in the deactivation of the catalyst. Higher temperatures can lead to catalysts sintering, increasing the formation of aromatics and coke, which leads to rapid deactivation of the catalyst [89].

### 3.3. Reaction Atmosphere

The catalytic deoxygenation reaction can be performed in an inert atmosphere, typically He and Ar, in a hydrogen atmosphere, or even in an H_2_/He or Ar mixture. The DO reaction can be greatly influenced by the type and the pressure of the gas used. Snare et al. conducted the reaction on different substrates (oleic acid, linoleic acid, and methyl oleate) over Pd/C, varying the reaction atmosphere. Pure H_2_, pure Ar, and H_2_–Ar mixture were used. Working in an H_2_-rich atmosphere, where the deoxygenation reaction is strongly promoted, they observed, for each substrate used, a greater conversion of hydrocarbons [90]. Similar results were obtained in the hydrotreating of ethyl stearate over Pd/C [91]. The authors observed that an H_2_-rich atmosphere promotes the hydrogenation of unsaturated species and therefore increases the concentration of saturated hydrocarbons in the biofuel. Kubickova et al. showed a lower number of aromatic hydrocarbons and a high concentration of saturated hydrocarbons in the H_2_ atmosphere, but more interesting, they also reported better conversion and catalyst TOF (turnover frequency) in 5%H_2_/Ar atmosphere [92]. In addition, the authors showed that *p*_H2_ affects the reaction; higher pressure leads to a lesser amount of unsaturated hydrocarbon compound. Particularly interesting results were reported by Santillan-Jimenez et al. in the DO of stearic acid and tristearin with Pd(5%)/C and Ni(20%)/C in pure H_2_, pure N_2_, and 10% miH_2_/N_2_ [93]. They observed different the catalysts’ activity depending on the atmosphere used; Ni/C is more promising in pure H_2_, while Pd/C is better in 10%H_2_/N_2_. In conjunction with the reaction atmosphere, *p*_H2_ can greatly influence the catalytic deoxygenation reaction. In methyl oleate, hydrotreatment over Pd/SBA-15, Lee and co-workers reported that an increase in pressure from 25 to 60 bar leads to a significant improvement in C_15_–C_18_ conversion and selectivity (up to 100% conversion and 70% selectivity C_15_–C_18_) [94]. A further increase from 60 to 80 bar leads to a decrease in conversion, and this is due to increased competition between the substrate and H_2_ for the catalyst’s active sites [94,95]. The positive effect of partial hydrogen pressure has also been reported by Nimkarde et al. in the DO of Karanja oil over NiMo and CoMo catalysts [96]. By increasing the pressure from 15 to 30 bar the conversion increased from 62.1 to 88.4% over NiMo and from 60.1 to 85.6% over CoMo. Sotelo-Boyas et al. observed a progressive increase in conversion and selectivity to HDO by increasing the pressure from 50 to 110 bar. A decrease in the percentage of heavy fractions in favor of light C_5_–C_12_ fractions, indicating that high pressures seem to favor a higher degree of cracking, was also observed [97]. This seems to be in contrast to what was observed by Yang et al. studying the DO of a mixture of C18 acids over sulfided NiW/SiO_2_-Al_2_O_3_. Varying the pressure from 20–80 bar, the C_3_–C_11_ light fraction yield decreased as the pressure increased, while the diesel yield increased up to 40 bar and then decreased at higher pressures [98]. The authors suggested that high pressure restrains cracking reactions and explained this with the Le Chatelier Principle. They also observed a decrease in DCO/DCO_2_ selectivity and a higher prevalence of HDO, at higher pressure, due to the major amount of hydrogen available for HDO. Higher pressure tends also to inhibit isomerization due a higher amount of H_2_ adsorbed on the catalyst sites used for isomerization. Anand et al. studied the DO of jatropha oil by varying the P from 20 to 90 bar [99]. Their work shows that an increase in conversion from 91 to 98% by increasing the pressure from 40 to 90 bar but a drastic reduction in conversion (31%) by working at pressures of 20 bar. Despite the high conversion obtained at 40 bar, the biofuel has a high concentration of oligomerized product (20% > C18), which decreases by increasing the pressure. The authors report that under their conditions the optimum pressure value is 80 bar.

### 3.4. Other Parameters

In addition to the previously discussed parameters, there are other variables that can influence the catalytic deoxygenation reaction such as the solvent, substrate, and reactor. Maki-Arvela et al. have observed a different reaction selectivity of their catalyst using free fatty acids (FFA) or methyl esters [91]. Using FFA, the reaction proceeds via DCO_2_, while using the corresponding methyl esters it appears that the reaction proceeds via DCO. In addition, the authors have also observed that compounds with longer chains tend to retard the reaction rate. Morgan et al., studying the DO of triolein and soybean oil under an inert atmosphere over a hydrotalcite-type catalyst, observed high cracking and coking activity only with soybean oil, which suggests that a higher degree of substrate unsaturation tends to favor cocking and cracking [100]. In addition, as observed by Kiatkittipong and co-workers, in the DO of CPO (crude palm oil), DPO (degummed palm oil), and PFAD (palm fatty acids distillate), the degree of substrate pre-treatment also seems to influence the reaction [101]. Using PFAD, the reaction requires less drastic conditions, and a better hydrocarbon yield is obtained. In addition, the authors also observed that Pd/C is more promising when working with PFAD, while sulfided NiMo/γ-Al_2_O_3_ is preferred with triglyceride-type substrates. The catalytic deoxygenation reaction can be performed in batch, semi-batch, or continuous reactors. Compared to continuous reactors, batch reactors allow for preliminary studies to be made to optimize reaction conditions and to generate kinetic data in an easy and economical manner [102]. The use of continuous and semi-batch reactors has the advantage of purging the reactor of CO_x_ formed during the reaction, and this has a dual advantage; one is to shift the balance of the reaction towards the products and the other is to avoid the poisoning of the catalysts by CO adsorption [95,103]. By comparing the same reaction conditions with the same catalyst (Pd/C), Snare et al. observed higher productivity in the semi-batch mode compared to the continuous reactor by attributing the cause to the mass transfer limitations in the fixed-bed reactor [90]. The solvent used can also slightly influence the catalytic deoxygenation reaction. Gosselink et al. evaluated the effect of the solvent by comparing *n*-decane, *n*-dodecane, and mesitylene and reported that *n*-decane and mesitylene led to better catalytic activity than *n*-dodecane [28]. Low-boiling solvents guarantee better activity [73,78]. The solvent can also modulate the activity of the catalysts, since Pt/C is more active than Pd/C in the DO of FFA in aqueous media while the opposite is the case in organic media [104,105].

## 4. Feedstock

Vegetable oils consist mainly of triglycerides with fatty acids, mostly unsaturated, with an alkyl chain in the range C_14_–C_18_ and also, in smaller quantities, longer alkyl chain fatty acids up to 22 carbon atoms [106,107,108,109]. The distribution and the type of fatty acids depend on the vegetable oil, as shown in Table 4 [37,110]. The distribution of fatty acids in each type of oil is not constant but may vary depending on the crop, environmental conditions, harvesting, and processing [107]. The choice of feedstock for biofuel production depends on several factors, such as commercial availability and oil yield from the seed [111]. Among the edible oils, the most important are palm oil, soybean oil, rapeseed (and canola) oil, and sunflower oil. America is the largest producer of soybean oil [112]. The oil content in soybean seeds is 18–21%, less than other oils such as sunflower oil and palm oil, while 38–44% are proteins. In fact, soybeans are grown mainly to produce meal [107]. Sunflower oil, on the other hand, is mainly produced in Europe and America, which account for about 50% of the world’s sunflower oil production [106,111]. Sunflower seed oil has 40–50% oil content [113]. Depending on the processing, it is possible to have oils with different fatty acid compositions, e.g., high stearic II sunflower oil has a higher concentration of stearic acid (35%) than conventional sunflower oil (5%) [106]. Palm oil is mainly produced in tropical regions such as Malaysia and Indonesia and is obtained in high yields from palm fruit or palm seed (called palm kernel oil) [108,114]. Together with coconut oil, palm oil is among the edible oils with the highest percentage of saturated fatty acids (Table 4).

Rapeseed oil and canola oil (a derivative of rapeseed oil) are mainly produced in China, Canada, India, and northern Europe [113]. Rapeseed oil is rich in erucic acid (which is harmful to human health) and has limited use as an edible oil. Therefore, its variant, canola oil, which is depleted of erucic acid, was developed [109]. For the DO process, the use of vegetable oils as saturated as possible is preferred because they reduce hydrogen consumption, allow the reaction to take place under less drastic conditions, and reduce selectivity towards undesirable reactions such as cracking, cyclization, and polymerization. Typically, vegetable oils with an iodine value (an experimental measure that determines the degree of unsaturation of a chemical species and is defined as the grams of iodine absorbed every 100 g of unsaturated species) ≤130 are preferred [115]. One of the problems related to the use of these vegetable oils for biofuel production is that most of them are edible, involving a competition between the biofuels sector and the food sector, so scientific research is particularly focused on the use of non-edible oils, such as jatropha oil, waste oils, or oils from microalgae [116]. A higher growth rate and triglyceride yield compared to many edible oils make microalgae a promising alternative to edible vegetable oils. They do not require arable land, can grow even with wastewater, and have an oil content in the range 20–50% of biomass weight [117]. Another non-edible oil is jatropha oil. Jatropha seeds can contain up to 60% oil. Jatropha oil does not compete with the food industry and the plant is particularly resistant to environmental conditions, which allows easy cultivation without hindering the land for food plant production [118]. The use of waste oil has several advantages, as it is an inexpensive oil since it is a waste material (in fact, it costs three times less than common vegetable oils) and reuse is environmentally friendly because, if not recycled properly, it becomes a polluting material [119]. Table 5 reports some examples of catalytic deoxygenation of vegetable oils.

### 4.1. Soybean Oil

Morgan et al. studied the DO of soybean oil under an inert atmosphere by comparing three different reduced metal catalysts supported on activated carbon, Pd 5 wt%, Pt 1 wt%, and Ni 20 wt% [44]. They observed that a higher metal content makes the Ni-based catalyst more active (leading to a 92% conversion) compared to noble metal catalysts. In contrast, 20 wt%Ni/C was also the catalyst with the highest cracking and methanation activity. The higher 20 wt%Ni/C activity is of particular interest from an economic point of view because noble metal catalysts have limited use due to their high cost. In addition, the authors observed an increase in the degree of unsaturation of the substrate, increasing the cracking reaction (soybean oil > triolein > tristearin). They also correlated the feedstock unsaturation degree with an increase in coke formation [100]. The effect of the percentage of supported metal has also been studied by Veriansyah and co-workers by evaluating a series of M/γ-Al_2_O_3_ catalysts (M = NiMo and CoMo sulfide, Pd, Pt, Ru reduced) and Ni/SiO_2_-Al_2_O_3_ reduced [29]. At catalyst-to-oil ratio = 0.088 and at high Ni contents in the catalyst (66.0 ± 3%), the Ni-based catalyst is among the most active, reporting a 96% conversion, deoxygenation > 90%, and a 99% in diesel selectivity. This may also partly depend on slightly different supports, but the authors do not report explanations for this. Working at a lower catalyst-to-oil ratio (0.044), the best catalyst is sulfided NiMo. Sulfided CoMo was instead the catalyst with the highest degree of hydrocracking, as it leads to a lower yield of organic liquid product and a higher percentage of light hydrocarbons. In addition to the active phase, the substrate used can also affect the activity and selectivity towards one hydrocarbon fraction rather than another. Zarchin et al. have used two Ni_2_P catalysts supported on SiO_2_ (neutral support) and HY (acidic zeolite) [120]. Both catalysts lead to an 82% yield of organic liquid products, but the composition is very different. The SiO_2_ catalyst only shows diesel hydrocarbon fraction selectivity, while with HY 40% of the liquid product is composed of a light fraction. The strong hydrocracking activity of this catalyst, however, is not stable over time; after 150h, there is a drop-in cracking activity in favor of greater diesel selectivity. The loss of cracking activity is attributed to the poisoning of the catalyst by CO and CO_2_ formed during the reaction. A similar effect of the support was also observed by Wang and co-workers evaluating the activity of NiMoC catalysts supported on different supports (Al-SBA-15, γ-Al_2_O_3_, ZSM-5, zeolite β, and USY) [70]. NiMoC/Al-SBA-15 is the most active catalyst, yielding 96% of liquid organic products and 97% in diesel selectivity, while zeolitic catalysts lead to a lower yield of the liquid organic product (60–80%) due to the higher degree of cracking and leading to a higher formation of hydrocarbons in the boiling range of green gasoline (15–40%).

**Table 5:** Catalytic deoxygenation reaction of different vegetable oils.

Feedstock	Reactor Type	Best Reaction Condition	Catalyst Form	Catalyst	Conversion%	Green Diesel Yield%	Ref.
Soybean Oil	Batch	400 °C, 92 bar H_2_, Cat/olio = 0.044–0.088, 2 h	Sulfided	NiMo/γ-Al_2_O_3_	92.9 (cat/oil = 0.044)	64.5% *n*-C_15_-C_18_	[29]
					91.9 (cat/oil = 0.088)	76.8% *n*-C_15_-C_18_	
				CoMo/γ-Al_2_O_3_	78.9 (cat/oil = 0.044)	33.7% *n*-C_15_-C_18_	
					79.9 (cat/oil = 0.088)	42.9% *n*-C_15_-C_18_	
			Reduced	Ru/γ-Al_2_O_3_	39.7 (cat/oil = 0.044)	32% *n*-C_15_-C_18_	
					Not tested (cat/oil = 0.088)		
				Pd/γ-Al_2_O_3_	91.9 (cat/oil = 0.044)	79.2% *n*-C_15_-C_18_	
					90.9 (cat/oil = 0.088)	72.6% *n*-C_15_-C_18_	
				Pt/γ-Al_2_O_3_	50.8 (cat/oil = 0.044)	37.7% *n*-C_15_-C_18_	
					Not tested (cat/oil = 0.088)		
				Ni/SiO_2_-Al_2_O_3_	60.8 (cat/oil = 0.044)	39.2% *n*-C_15_-C_18_	
					95.9 (cat/oil = 0.088)	74.8% *n*-C_15_-C_18_	
	Batch	350 °C, 10 bar H_2_, Cat/olio = 25 wt%, 5 h	Calcined	NbOPO_4_	100	40% Green Diesel	[121]
	Flow	325–360 °C, 50 bar H_2_, H_2_/feed = 1800 mL/mL, WHSV ^1^ = 7 h^−1^	Sulfided	NiMo/USY ^2^-Al_2_O_3_	100 (360 °C)	81.9% *n*-C_15_-C_18_	[122]
				NiMo/HY ^3^-Al_2_O_3_	100 (360 °C)	68.8% *n*-C_15_-C_18_	
				NiMo/β ^4^-Al_2_O_3_	100 (360 °C)	5.7% *n*-C_15_-C_18_	
				NiMo/ZSM-5 ^5^ (90)- Al_2_O_3_	100 (360 °C)	0 *n*-C_15_-C_18_	
				NiMo/ZSM-5(1770)-Al_2_O_3_	100 (360 °C)	79.5% *n*-C_15_-C_18_	
				NiMo/Al_2_O_3_	100 (360 °C)	81.5% *n*-C_15_-C_18_	
	Flow	357 °C, 40 bar H2, ^10^ LHSV = 1 h^−1^, H2/oil = 1765 *v*/*v*.	Reduced	Pt/SAPO-11 ^6^-Al_2_O_3_	100	79.8% hydrocarbon fuel (63.3 *i*-Alkane selectivity)	[35]
				Pt/ZSM-22-Al_2_O_3_	100	49.8% hydrocarbon fuel (84.3 *i*-Alkane selectivity)	
	Flow	400 °C, 45 barH_2_, LHSV = 1 h^−1^, H_2_flow = 50 mL/min	Reduced	NiMoC/Al-SBA-15	100	~97% Green Diesel	[46]
				NiMoC/γ-Al_2_O_3_	100	~85% Green Diesel	
				NiMoC/ZSM-5	100	~28% Green Diesel	
				NiMoC/Zeolite β	100	~32% Green Diesel	
				NiMoC/USY	100	~55% Green Diesel	
Rapeseed oil	Flow	360°C, 70 bar H_2_, H_2_ flow = 0.1 Nm^3^/h, feed flow = 100 g/h WHSV = 1 h^−1^, H_2_/oil = 920 Nm^3^/m^3^	Commercial	NiMo/Al_2_O_3_	>99%	24.9% *n*-C_17_ 51.8% *n*-C_18_	[123]
	Flow	350 °C, 31 bar H_2_, H_2_/oil = 600 mL/mL, LHSV = 1 h^−1^	Sulfided	NiMo/γ-Al_2_O_3_	100	69% *n*-C_15_-C_18_	[124]
				CoMo/γ-Al_2_O_3_	>95%	68.% *n*-C_15_-C_18_	
Palm Oil	Flow	380 °C, 40 bar H_2_, WHSV = 2 h^−1^, H_2_/oil = 2370 Ncm^3^/cm^3^	Reduced	Pt/γ-Al_2_O_3_	100	75.2% *n*-C_15_-C_18_	[125]
			Phosphided	Ni_2_P/SiO_2_	100	76.3% *n*-C_15_-C_18_	
				Ni_2_P/γ-Al_2_O_3_	100	79.7% *n*-C_15_-C_18_	
	Flow	425 °C, 50 bar H_2_, H_2_/oil = 1000 *v*/*v*, WHSV = 0.9 h^−1^	Reduced	Ni_2_P-Ni_12_P_5_/NaMOR ^7^	100	83.5% *n*-C_15_-C_18_	[126]
	Flow	300 °C, 50 bar H_2_, LHSV = 2 h^−1^, H_2_/oil = 1000 N cm^3^/cm^3^	Reduced	NiAl_2_O_4_ spinel-type	100	94% *n*-C_15_-C_18_	[127]
	Batch	370 °C, 40 bar H_2_, Cat/oil = 0.5 g/25 cm^3^	Sulfided	ReNiMo/γ-Al_2_O_3_	100	72.5–69.5% C_13_-C_18_	[128]
	Batch	285 °C, 10 bar N_2_, 80 g H_2_O, cat/oil = 0.046 (20 g oil, 0.92 g cat)	Reduced	Pt-Re/CNT ^8^	-	72% *n*-C_15_-C_18_	[129]
Sunflower Oil	Flow	380 °C, 20–80 bar H_2_, LHSV = 1 h^−1^, H_2_/oil = 600 Nm^3^/m^3^	Reduced	NiMo/Al_2_O_3_	100	69.5–73.1% C_11_-C_19_	[62]
				CoMo/Al_2_O_3_	65.1–74.9	44.8–50.4% C_11_-C_19_	
	Semi-Batch	310 °C, 40 bar H_2_, H_2_ flow = 100 mL/min, oil/cat = 100 mL/g	Reduced	NiMo/Al_2_O_3_ (three different synthesis)	99	97% C_15_-C_18_	[130]
	Semi-Batch	310 °C, 40 bar H_2_, H_2_ flow = 100 mL/min, oil/cat = 100 mL/g	Reduced	NiZn/Al_2_O_3_ (three different NiZn wt%)	99	72% C_15_-C_18_	[131]
	Flow	400 °C, 180 bar H_2_, sunflower flow = 49 gh^−1^, H_2_ flow = 0.049 Nm^3^h^−1^	Sulfided	Sulfided commercial hydrocracking catalyst	100	18.1% *n*-C_15_-C_18_ 63.3% *i*-alkanes + cycloalkanes	[132]
Jatropha Oil	Flow	370 °C, 35 bar H_2_, LHSV = 0.9 h^−1^, H_2_/oil = 1000 mL/mL	Reduced	NiMoLa(X)/Al_2_O_3_ (X = 0.5–15 wt%)	83	78% *n*-C_15_-C_18_	[133]
	Flow	370 °C, 35 bar H_2_, LHSV = 0.9 h^−1^, H_2_/oil = 1000 mL/mL	Reduced	Reduced NiMoCe(X)/Al_2_O_3_ (X = 0.5–15 wt%)	89	80% *n*-C_15_-C_18_	[134]
Microalgae Oil	Batch and Flow	260 °C, 40 bar H_2_, Cat/oil = 0.2, dodecane	Reduced	Ni/HBeta ^9^	100	72% *n*-C_15_-C_18_	[77]
	Batch	260 °C, 40 bar H_2_, Cat/oil = 0.2, dodecane	Reduced	Ni/HBeta	100	71% C_15_-C_18_	[116]

^1^ WHSV = weight hourly space velocity; ^2^ USY = ultrastable zeolite Y; ^3^ HY = hydrogen form of zeolite Y; ^4^ β = zeolite beta; ^5^ ZSM-5 = zeolite Socony Mobil-5; ^6^ SAPO-22 = Santa Barbara amorphous-22; ^7^ NaMOR = sodium form of zeolite mordenite; ^8^ CNT = carbon nano tubes; ^9^ HBeta = hydrogen form of beta zeolite; ^10^ LHSV = liquid hourly space velocity.

In fact, zeolitic catalysts are widely used to produce biofuels in the jet and gasoline range due to their cracking activity. Wang et al. have also studied NiMo/ZSM-5 carbide and nitride catalysts to compare the activity towards the hydrocracking of soybean oil to obtain hydrocarbons in the gasoline range [75]. They observed that the carbide catalyst is better than the nitride catalyst, for their purpose, because it leads to a higher gasoline selectivity and a lower methanation reaction; they also observed the beneficial effect of Ni as a promoter in Mo-based catalysts. Indeed, the catalyst only containing Mo led exclusively to high molecular weight compounds (c > 23). Working in a continuous flow reactor, they also studied the effect of LHSV, observing that increasing LHSV decreased the yield of the liquid product and increased the selectivity to gasoline range hydrocarbon. Too high LHSV led to products derived from oligomerization (high LHSV promotes higher concentrations of unsaturated compounds that are more prone to oligomerization reactions). Correlation between the support acidity and cracking reaction was observed by Zandonai and collaborators by conducting the catalytic deoxygenation of soybean oil with ZSM-5 in its protonated form, HZSM-5, and in the NaZSM-5 form [112]. It was evident from the work that the more acidic support HZSM-5 leads to increased hydrocarbon formation and selectivity towards the gasoline range. For green diesel production a certain degree of cracking is desirable, as it promotes the isomerization reaction leading to a biofuel with better cold properties. Pt and Pd-based catalysts supported on SAPO-11, a zeolite that combines moderate acidity with a particular cavity geometry that makes it suitable for isomerization, have a slight degree of cracking that makes them valid candidates for hydroisomerization reactions. These supports, however, suffer from a severe deactivation. In fact, even the hydrotreating of a mixture of paraffins with 5% fatty acid (oleic acid) leads to a rapid loss of isomerization activity due to H_2_O that forms during deoxygenation and hydrolyzes part of the support [135]. In this regard, Rabaev et al. evaluated the activity of a Pt-based catalyst supported on a modified SAPO-11 with the addition of alumina (Pt/SAPO-11-Al_2_O_3_), observing that the addition of alumina leads to higher hydrothermal stability but also to higher acidity and isomerization capability [136]. With this catalyst, the authors obtained a 99% deoxygenation of soybean oil and a liquid organic product with a cloud point < −35. Similar work has also been reported previously [137]. Given the correlation between cracking and isomerization, an increase in temperature and in catalyst acidity leads to an increase in *i*-alkane percentage [35].

### 4.2. Rapeseed Oil

Kubicka and Kaluza demonstrated the efficiency of Ni as a promoter in Mo-based catalysts, evaluating the different activity between sulfided mono-metallic Ni and Mo catalysts and sulfided NiMo (at different NiMo ratios) supported on γ-Al_2_O_3_ [39]. Bimetallic catalysts are more active and selective than monometallic catalysts. They present a higher conversion rate and a higher degree of deoxygenation; at the same conversion degree, they have a higher selectivity towards the formation of hydrocarbons. The different Ni/Mo ratio does not seem to particularly affect the activity of the catalyst. Due to mild reaction conditions, no cracking and isomerization products were observed. They also observed that Ni prefers DCO/DCO_2_, Mo prefers HDO, and NiMo gives both reactions to the same extent. Simacek et al. evaluated the effect of temperature and a sulfided NiMo/γ-Al_2_O_3_ catalyst (varying Ni and Mo content) on the DO of rapeseed oil [138]. They noted that a temperature of at least 310 °C is required to obtain a liquid organic product free of starting intermediates and triglycerides. The amount of metal in the catalyst does not particularly affect the activity. However, they observed that the catalyst with the highest Mo/Ni ratio is the one that leads to a higher amount of isomer hydrocarbons, especially at higher temperatures. Catalysts prefer an HDO reaction, but as the temperature increases, there is an increase in DCO/DCO_2_ products. The supported active phase plays a fundamental role in reaction selectivity and reaction activity, and in this regard, Zhang and collaborators have carried out a study on the different behaviors of unsupported CoMoS and NiMoS (excluding the effect of the support) in the DO of rapeseed oil [139]. Both catalysts exhibit complete conversion, but NiMo has a higher conversion rate, higher diesel selectivity (less cracking), and a liquid product consisting of 90% n-alkanes. On the other hand, CoMo leads to more cracking and the liquid product is richer in olefins, indicating a lower hydrogenation capacity. The lower hydrogenation capability agrees with the fact that CoMoS prefers DCO/DCO_2_ while NiMoS prefers HDO. The authors report that the different behavior of the two catalysts is due to the structure of the active phase. In fact, NiMo is richer in sulfur vacancies and therefore has principally unsaturated active sites, while with CoMo saturated sites dominate, and this leads to a different selectivity of the reaction. Similar work has been addressed by Priecel et al. by analyzing the effect of Ni on the activity of the NiMoS catalyst. It is reported that the effect of Ni is related to the geometric structure it assumes in the catalyst (in this case depending on the calcination temperature used during the catalyst synthesis), which influences its ability to be activated by sulfurization [140]. An issue in the use of sulfided catalysts is their instability. Sulfided catalyst deactivation has been studied by Kubicka and Horaceck by evaluating the activity of sulfided CoMo/γ-Al_2_O_3_ in the DO of several rapeseed oils with different degrees of upgrading [41]. They observed that the presence of alkalis causes deactivation by depositing on the catalyst and inducing electronic effects on the active phase, which inhibits its activity [141]. When not counterbalanced, phosphates also cause deactivation as in the reaction environment they form phosphoric acid, which promotes polymerization reactions by forming high-weight compounds that adsorb on the catalyst, inhibiting its activity. However, the authors attribute the main cause of deactivation to sulfur leaching because the addition of a sulfuring agent to the substrate limits the deactivation of these catalysts. They also noted that the addition of a sulfur agent alters the reaction selectivity from mainly HDO to an average between HDO and DCO/DCO_2_. Obviously, the use of sulfur catalysts combined with the addition of sulfur sources inevitably leads to contamination of the product, and this is the major drawback in the use of sulfur catalysts. An alternative to sulfided catalysts can also be nitride catalysts, as observed by Monnier and collaborators studying the activity of nitride catalysts MN_x_/γ-Al_2_O_3_ (M = Mo, W, V) in canola oil deoxygenation [74]. The most active catalyst was MoN_2_ with 100% of conversion, a liquid product yield of 84.1 wt%, and 30 wt% *n*-alkanes. Mo prefers HDO while the other prefers DCO/DCO_2_. Therefore, Mo has the highest hydrogenation activity. Long-term experiments have shown that MoN_2_ is stable up to 450 h, obtaining 48 g of organic liquid product in the diesel boiling range every 100 g of oil. MoN_2_/γ-Al_2_O_3_ is, however, less active than NiMoS/γ-Al_2_O_3_, from which a hydrocarbon yield in the diesel boiling range of 80 g/100 g of the substrate and a much lower formation of high distillation products is obtained. In addition to the active phase, it is also the support that plays a fundamental role in the DO process. Kubicka et al. investigated the support effect (SiO_2_, γ-Al_2_O_3_, and TiO_2_) in Ni(3.3 wt%)Mo(5 wt%)S bimetallic catalysts in rapeseed oil DO [142].

For all the tested catalysts, there is 100% conversion at 300 °C, but the highest degree of deoxygenation can be achieved with SiO_2_, which has a larger surface area, higher acidity, and greater dispersion of the active phase. The authors observed that the support can also influence reaction selectivity because SiO_2_ catalysts favor DCO/DCO_2_ by attributing this preference to a greater dispersion of the active phase, stronger interaction between the active phase and support, the high metal’s loadings, and a larger pore diameter that is more suitable for large molecules such as triglycerides. The efficiency of catalysts based on mesoporous supports was discussed in another work by Kubicka and co-workers comparing the activity of CoMoS/OMA (OMA = organized mesoporous alumina), CoMo/MCM-41, and CoMo/γ-Al_2_O_3_ (all CoMo catalysts have 3 wt% Co and 15 wt% Mo) in the DO of rapeseed oil [143]. Their study shows that OMA is more active than γ-Al_2_O_3_ catalysts. In contrast, the Si-based support (MCM-41) is the least active (OMA > γ-Al_2_O_3_ > MCM-41). The authors correlate the lower activity of MCM-41 compared to OMA with a more unfavorable interaction between the active phase and the Si-based support than alumina-based support. The liquid product obtained is also free of light fractions and aromatics. This is related to the higher diffusivity guaranteed by the mesoporous support and the not too high reaction temperature (310 °C). The same authors have carried out a similar study confirming that MCM-41 supports are not as active as classic alumina-based catalysts but that the introduction of Al in the MCM-41 support improves the activity of the catalyst, demonstrating the beneficial effect of Al [30]. The support effect also affects the selectivity of the hydrocarbon fractions produced. Sotelo-Boyas et al. analyzed the activity of catalysts that have different characteristics, namely NiMoS/γ-Al_2_O_3_ (typical hydrotreating catalyst) and Pt/zeolite (zeolite = HZSM-5 and HY typical hydrocracking catalysts) [97]. NiMo/γ-Al_2_O_3_ gives a high diesel selectivity, while Pt/zeolite catalysts give a high degree of cracking and therefore a higher selectivity for green gasoline. With the NiMo catalyst they observed that the highest yield in terms of liquid products (86%) and selectivity in hydrocarbons (78%) is obtained at 350 °C and *p* > 80 bar. Although the authors work under conditions similar to those of Simacek et al. [138], the reaction needs more drastic conditions, and this is attributed to the different reactor used (batch vs. continuous reactor). The reaction conducted with Pt/Zeolite catalysts requires more severe conditions to obtain liquid products free of oxygenated compounds (380 °C and 110 bar). Comparing the Pt/Zeolite catalysts, they observed that HZSM-5 (zeolite is more acidic than HY) gives a higher gasoline yield (40% green gasoline) and a lower diesel yield, while HY is the opposite, making it a more suitable catalyst for the synthesis of green diesel with a certain degree of isomerization. HZSM-5 is more suitable for green gasoline synthesis.

### 4.3. Palm Oil

Palm oil is among the vegetable oils with the highest content of saturated fatty acids, and this can influence the DO reaction as observed by Guzman et al. in the study of palm oil deoxygenation with NiMoS/γ-Al_2_O_3_ in a pilot plant scale [144]. They observed that as the pressure increased, there was a higher content of n-alkanes and a lower presence of isomer species. At P = 90 bar, the presence of aromatic compounds is not observed differently from what was observed in Da Rocha et al.’s work, even at 140 bar [145]. The authors explain this difference in the different oil used in the two works; oils richer in unsaturation (such as the soybean oil used by Da Rocha et al.) are more susceptible to aromatization reactions. The catalyst exhibits a progressive deactivation over time, but even after 14 h, a liquid product with a satisfactory cetane number was obtained. Reduced metal catalysts can be a good alternative to classic sulfided catalysts. Srifa et al. have examined the activity of a Ni/γ-Al_2_O_3_ and Co/γ-Al_2_O_3_ (both 10 wt%) reduced catalyst in the DO of palm oil [146]. For both catalysts, they observed 100% conversion up to 150 h, but after 100 h, there was a decrease in the organic product yield from 92.2 to 75.6% for Ni and from 88.6 to 56.6% for Co. The analysis of the recovered catalysts shows a certain degree of sintering, but the deactivation after 100 h is attributed to the coke formation because through proper treatment that leads to the removal of coke. The authors found that the catalyst fully recovers its textural properties, which suggests a complete regeneration of catalytic performance. In another work, Srifa and co-workers compared the activity of different M/γ-Al_2_O_3_ catalysts, where M = Co, Ni (5–10 wt%), Pd, and Pt (2–5 wt%), in the hydrotreatment of palm oil [147]. For higher metal loading (5–10 wt%%), a conversion of 100% is observed, and at the same charged metal (5 wt%) the yield in terms of liquid organic products follows the trend Co (88.5%) > Pd (85.2%) > Pt (79.5%) > Ni (69.7%). Ni, Pd, and Pt prefer DCO/DCO_2_, while Co prefers HDO. Wang et al. analyzed the effect of tungsten as a promoter in a NiMoW/γ-Al_2_O_3_ catalyst; the effect of a 15 wt% addition of ZSM-5 in γ-Al_2_O_3_ was also studied [148]. From the catalysts’ screening, the authors observed that the NiMoW/γ-Al_2_O_3_-ZSM-5 catalyst was the most active, leading to high diesel selectivity. This higher activity is attributed to the improved acidity of this catalyst (given by the addition of ZSM-5) but also by the promoter effect of W. In fact, comparing NiMoW/γ-Al_2_O_3_ in the absence and in presence of W, it was observed that besides NiMoW/γ-Al_2_O_3_ -ZSM-5 the most active catalyst is NiMoW/γ-Al_2_O_3_. The higher acidity of the modified support also leads to a higher degree of isomerization, obtaining a liquid product with a good cetane value (66) and cloud point (−5 °C). The support’s effect on isomerization activity was also studied by Liu and co-workers using a Ni-based catalyst (7 wt% Ni) supported on three nano-sized SAPO-11 zeolites with different particle sizes [149]. The best liquid hydrocarbon yield (80%) and isomerization selectivity (81.2%) were obtained with the support having the largest surface area and mesoporous volume. The synthesized SAPO-11 also has higher activity and stability compared to the commercial SAPO-11, giving a higher yield of liquid alkanes and i/n selectivity. The improved stability is attributed to the higher mesoporous volume and the greater dispersion of the metal on the synthesized support, which favors the diffusion of reagents and products minimizing coke formation. Smaller particle sizes increase the support surface area, promoting the diffusion of particularly large reagents such as triglycerides [150]. In another work, Liu et al. have analyzed the effect of metal loading (Ni 2, 5, 7, 9 wt%) on their formulation of SAPO-11 with a larger surface area and smaller particle size [88]. The authors observed that as the Ni content increases, liquid hydrocarbon yield and isomerization selectivity increases. However, the cracking also increases. At 360 °C, 40 bar H_2_, TOS = 6 h, and LHSV = 1 h^−1^ the 7% formulation results in a good compromise that leads to a yield in liquid hydrocarbons of 67.4%, high selectivity of isomerization (61.5%), and a negligible cracking. As the temperature increases, the isomerization selectivity also increases, but too high temperatures also result in high cracking activity. The catalyst at 7% is stable up to 35 h, observing a low coke formation favored, as already stated, by the mesoporous support. The authors suggest that the support’s medium strength acid sites are responsible for the high isomerization activity.

### 4.4. Sunflower Oil

Studies on sunflower oil are less common than those with other vegetable oils. Krar et al. evaluated the effect of reaction conditions in sunflower oil deoxygenation with reduced CoMo/γ-Al_2_O_3_ [61]. They observed that in order to obtain a 100% conversion it is necessary to work at 380 °C. As the temperature increases, the yield of the product decreases, and the yield of the diesel fraction increases. Increasing LHSV increases the yield of liquid organic products but decreases the yield of the diesel fraction (it seems that pressure has no effect at this reaction temperature). The best reaction conditions are 380 °C, 40 = −60 bar H_2_, H_2_/oil = 500–600 *v*/*v*, LHSV = 1.0 h^−1^. In any case, the catalyst prefers HDO reaction, but as the temperature increases, the formation of DCO/DCO_2_ products increase; the same trend is observed if the pressure decreases. The authors also made a comparison between the reduced and sulfided catalysts. They suggest that the reduced catalyst is more convenient because it does not require the addition of sulfur agents, which is necessary to maintain the sulfur catalysts activity; the yield of the diesel fraction is only 5% lower. Another comparison between reduced and sulfided catalysts was reported by Harnos and co-workers. They studied the deoxygenation of sunflower oil with NiMo/γ-Al_2_O_3_, Ni/γ-Al_2_O_3_, Pd/C, and Pd/γ-Al_2_O_3_ catalysts [45]. Among the bimetallic catalysts, a sulfided catalyst is more active with a liquid product yield of 73% (n-C_17_ = 26.3% and nC_18_ = 28.1%), while reduced NiMo leads to a liquid product yield of 66% (n-C_17_ = 5.1% and nC_18_ = 45.9%) with a higher degree of methanation (therefore higher H_2_ consumption). They noted that reduced Ni/γ-Al_2_O with a 27 wt% Ni content has similar activity as the sulfided NiMo catalyst and therefore represents an alternative to sulfided catalysts. The authors also evaluated the effect of the reaction gases on the stability of the catalyst and concluded that they have no particular effect, suggesting the possibility of using the syngas as a source of H_2_ or reusing the recycled gas of a run for subsequent DO reactions. In other cases, it has been observed that a slight modification of the support leads to better catalyst activity. Duan et al. have studied the effect of Al incorporation in Pd/Al-SBA in the DO of sunflower oil [151]. The catalyst with the highest Al content is the most active, probably due to the enhanced acidity provided by the higher Al content, and this is in agreement with the observations made by Kubicka et al. [30]. The authors also observed that as acidity increased, selectivity towards HDO increased, suggesting a correlation between acidity and the HDO reaction. The effect of temperature was also evaluated, suggesting that the optimal temperature is 250 °C, because at higher temperatures there is excessive cracking while at lower temperatures the product is a partially solid index of a high presence of oxygenated compounds. Kikhtyanin et al. have examined the DO of sunflower oil over 1% Pd/SAPO31 (typical hydroisomerization catalyst) in order to obtain a biofuel in the diesel range and with good cold properties [152]. Only for T > 320 °C and WHSV = 0.9–1 h^−1^ the liquid product obtained is free from oxygenated intermediates. As the temperature increases, DCO/DCO_2_ and isomerization reactions are more favored. From the stability test at 340 °C, 20 bar H_2_, and WHSV = 0.9 h^−1^ the catalyst remains stable for long periods with an i/n value of about 10 and a liquid product that consists of 99% hydrocarbons; with higher WHSV there is a drastic decrease in hydroisomerization activity but not in deoxygenation capability of the catalyst. The deactivation of the isomerization is attributed to the sintering of the catalyst. At 340 °C, 20 bar H_2_, and WHSV = 0.9 h^−1^ the liquid product has a cloud point value of about −50 °C. Dominguez-Barroso et al. conducted an interesting study on sunflower oil deoxygenation in an H_2_ free environment, under sub-critical H_2_O conditions over PtNi/Al_2_O_3_, and Pd/C combined catalysts [153]. They observed that, under operating conditions, the non-catalytic reaction allows the complete hydrolysis of triglycerides only at H_2_O/oil ratio = 2 ratio. Using PtNi/Al_2_O_3_ and Pd/C combined catalysts results in an 86% conversion to hydrocarbon with a diesel range (C_10_–C_20_ alkanes) selectivity of 90.7% C_10_–C_20_. Finally, the authors proposed that the glycerol formed as a result of triglyceride hydrolysis can undergo aqueous phase reforming (APR), which forms H_2_ in situ to be used in the DO process.

### 4.5. Non-Edible Oil

Toba et al. studied the DO of waste oils over sulfided CoMo, NiMo, and NiW catalysts supported on mesoporous Al_2_O_3_ [53]. All catalysts tested yield a high degree of deoxygenation (T ≥ 300 °C), but with CoMo the product obtained contains high olefin concentration (lower hydrogenation capacity). They also tested the activity of NiMo/B_2_O_3_-Al_2_O_3_, observing that the higher acidity provided by B_2_O_3_ increases the formation of iso-alkanes. By conducting the reaction with different waste oils (richer in free fatty acids) the authors observed that a different content of free fatty acids does not influence catalyst activity. At 350 °C all catalysts used are stable for 24 h, but at 300 °C there is a higher deactivation caused by more oxygenated compounds adsorbing on the catalyst. The composition of the liquid obtained with NiMo and NiW remains constant in time, while with CoMo it varies due to a more extensive deactivation (due to a greater formation of olefins). The activity of sulfided NiMo/Al_2_O_3_ and CoMo/Al_2_O_3_ has also been studied by Garcia-Davilla et al., but in this case, they used jatropha oil as the feedstock [154]. They observed that as the temperature increases the conversion and the percentage of hydrocarbons in the liquid product increase. At 390 °C NiMo is the most active catalyst, leading to a conversion of 65% and a liquid product consisting of 52 wt% hydrocarbons with a selectivity of 70% in the diesel range, 25% jet fuel range, and 5% kerosene range. In addition, the authors noted that an increase in residence time leads to a higher diesel fraction. The higher activity of NiMo is attributed to its higher acidity than CoMo. The catalyst’s effect on the reaction selectivity was analyzed by Tiwari et al. by conducting the deoxygenation of waste soybean oil and refinery-oil mixture over a sulfided NiMo/Al_2_O_3_ hydrotreating catalyst and sulfided NiW/SiO_2_-Al_2_O_3_ hydrocracking catalyst [55]. Both catalysts lead to complete conversion; NiMo has high diesel selectivity (85–95%), while NiW has higher kerosene selectivity (50–15%). The reaction selectivity reports that NiW prefers DCO/DCO_2_, while NiMo prefers HDO. In this case, the authors also analyzed the sulfur content in the liquid product obtained, observing a higher percentage of sulfur removal for NiMo (86–93%) and lower for NiW (60–85%). Considering that deoxygenation is complete in both cases, the authors suggest that HDO is more favored than HDS. Similar work has been addressed by Bezergianni et al. by conducting catalytic deoxygenation of waste oils by testing the activity of three commercial sulfided catalysts, a NiMo catalyst (A) for hydrotreating, a CoMo catalyst (B) with mild hydrocracking capacity, and a NiMo catalyst (C) with high hydrocracking activity [155]. Maximum conversion (90% at 330 °C) and maximum diesel selectivity (79% at 370 °C) are obtained with catalyst A. Lower conversions were obtained with the other two catalysts, even at higher temperatures. The liquids obtained were analyzed by simulated distillation; the distillation curve obtained with catalysts A and C shows that 95 wt% of the liquid boils at T< 530 °C (temperature at which 95 wt% of the waste oil boils), while for the liquid obtained with B the distillation curve shows the presence of high molecular weight compounds. The catalysts A and C also show the best activity in the removal of heteroatoms (O, S, N), and also the liquid products of A are those with less unsaturated compounds, which also indicates a higher hydrogenation activity of catalyst A. Peng and co-workers studied the DO of microalgae oil over Ni supported on five different supports, namely ZrO_2_, TiO_2_, CeO_2_, Al_2_O_3_, and SiO_2_ (with different metal loading 5, 10, and 15 wt%) [52]. The authors observed that at low metal content there is a lower selectivity in hydrocarbons and a large presence of oxygenated compounds. The best catalyst was found to be Ni/ZrO_2_, leading to a conversion of 100% and 76 wt% of liquid hydrocarbon (for 10–15 wt% metal loading) with the prevalence of n-C_17_ alkane (68 wt%). Stability tests show that the catalyst was found to be active for 72 h without variation in the composition of the product. The authors also observed that the formation of *n*-C_17_ also increases with increasing pressure (from 26 to 68%), but at pressures above 40 bar the concentration of *n*-C_17_ decreases as HDO is favored. Meller and co-workers studied the DO of castor oil FAME with commercial Pd/C, also evaluating the effect of the solvent by conducting the reaction in supercritical n-hexane and *n*-dodecane [156]. The high viscosity of castor oil FAME leads to engine issues, so it is useful to convert them into hydrocarbons via a deoxygenation reaction. The authors observed that the reaction conducted in supercritical n-hexane leads to higher hydrocarbon yield (57%) than the reaction conducted in n-dodecane (39.6%). The higher yield obtained with n-supercritical n-hexane is attributed to a higher diffusivity of reagents and products. As the temperature increases, there is an increase in hydrocarbons content in the product; at 340 °C the liquid product obtained is free of oxygenated compounds and consists of 96% alkanes distributed in 87% heptadecane and 9% octadecane (preference for DCO/DCO_2_). In their case, the pressure does not seem to influence the hydrocarbon yield. Verma et al. investigated the DO of jatropha oil using two sulfided NiMo and NiW catalysts supported on hierarchical mesoporous SAPO-11 (at different Si/Al values) [84]. The authors are interested in cracking, so the reaction is conducted at higher temperatures (374–450 °C). At lower temperatures, the catalysts lead to a liquid product with a higher diesel selectivity, but at higher temperatures, there is an increase in the kerosene range. At 425 °C there is the maximum kerosene yield (22–37.5%). A similar trend occurs as the pressure increases. The variation of Si/Al does not seem to have a particular effect, except in the case of their NiW/MSP-2 formulation, which has a higher acidity and leads to a 30% increase in kerosene yield compared to its counterpart with less acidic support. Furthermore, the authors observed that the i/n ratio increases with increasing temperature and decreasing pressure, while the aromatic content increases with increasing temperature and pressure (up to 8%). Li and co-workers analyzed the DO of waste oils for jet fuel production using Ni (10 wt%)/zeolite hydrocracking catalysts (zeolite = SAPO-11, HY, Meso-Y) [83]. HY and MESO-Y have higher jet fuel selectivity, while SAPO-11 leads to higher diesel selectivity (lower acidity of SAPO-11 than HY and MESO-Y leads to higher diesel selectivity). The authors recommend the use of Ni/MESO-Y, as it leads to a higher jet fuel yield and a content of aromatic compounds suitable for jet fuel specifications (HY leads to too high a number of aromatics). At 400 °C and 30 bar H_2_ (optimized reaction conditions), Ni/MESO-Y leads to a biofuel with good jet fuel selectivity (40.5%) and 11.3 wt% aromatic compounds.

## 5. Conclusions

The catalytic deoxygenation reaction of vegetable oils is an innovative process for producing hydrocarbon biofuels that are able to successfully replace petroleum derivatives. The triglycerides of vegetable oils are converted into hydrocarbons through three main reactions: decarbonylation (DCO), decarboxylation (DCO_2_), and hydrodeoxygenation (HDO). The type of hydrocarbons and therefore the type of biofuel that can be obtained depends on numerous factors such as temperature, hydrogen pressure, catalyst, and the type of substrate used. It is generally reported that the most active catalysts are NiMo, CoMo, NiW sulfide catalysts, or noble metal catalysts, supported on materials such as Al_2_O_3_, TiO_2_, ZrO_2_, and AC. However, reduced catalysts of transition metals have also been developed, and they can be, when properly prepared, more active than sulfided and noble metal ones. Moderately acidic supports seem to favor the catalytic deoxygenation reaction without leading to high coke formation, but recently, numerous applications in mesoporous substrates were reported, as they guarantee a more efficient diffusion of the reagents, limiting cracking and coke formation. Temperature and hydrogen pressure also play an important role. In fact, higher temperatures tend to increase conversion, increasing cracking, while a moderate pressure of hydrogen seems to be necessary for a good conversion of the substrate and to keep the catalyst active. Compared to model compounds, the use of vegetable oils generally requires harsh reaction conditions and often leads to a lower formation of coke and cracking due to the unsaturation present. The use of oils that are as saturated as possible, which also translates into savings in hydrogen consumption, is recommended. The choice of vegetable oil, however, is often guided essentially by territorial availability. Due to the competition with the food industry, the scientific community is focusing on the use of exhausted or non-edible vegetable oils. In conclusion, the catalytic deoxygenation process is a useful tool to produce hydrocarbon biofuels that are able to replace the common fuels derived from oil, and the main goal of the scientific community is to investigate more active and economical catalysts, less drastic conditions such as lower temperatures and pressures, and waste or non-edible feedstock to make this process more economical and environmentally friendly.

## Figures and Tables

**Table 1:** Increase in global energy demand from 2018 to 2040.

		Mboe/d ^1^			Growth % (Mboe/d)	Share of Global Energy Demand (%)			
	2018	2020	2030	2040	2018–2040	2018	2020	2030	2040
OECD ^2^ Countries	110.6	111.2	110.5	105.5	−3.1	38.7	37.9	33.6	30.1
Non-OECD Countries	175.3	182.2	218.4	250.1	+74.8	61.3	62.1	66.4	69.9
World	285.9	293.4	328.9	357.5	+71.7	100	100	100	100

^1^ Mboe/d: millions of barrel oil equivalent; ^2^ OECD: Organization for Economic Co-operation and Development.

**Table 2:** Physical properties of petrol diesel, biodiesel, and green diesel (Figure modified from [23]).

Properties	Petrol Diesel	Biodiesel	Green Diesel
Cetane Number ^1^	40	50–65	70–90
Energy Density (MJ/kg)	43	38	44
Density (g/mL)	0.84	0.88	0.78
Sulfur (ppm)	<10	<1	<1
Cloud Point (°C)	−5	−5, +15	−20, +20
Oxidative Stability	Good	Marginal	Good
Cold Flow Properties	Good	Poor	Poor

^1^ Cetane number is an indicator of the combustion speed and compression needed for ignition of diesel fuel.

**Table 4:** Fatty acid distribution of different vegetable oils.

Vegetable Oil Composition (%wt)									
Fatty Acid	Soybean	Rapeseed	Palm	Sunflower	Peanut	Corn	Jatropha	Canola	Microalgae
Lauric (C12:0) ^1^	0.0	0.0	0.1	0.0	0.0	0.0	0.0	0.0	0.0
Myristic (C14:0)	0.0	0.0	0.7	0.0	0.1	0.0	0.0	0.1	0.6
Palmitic (C16:0)	11.3	4.9	36.7	6.2	8.0	6.5	15.9	5.1	27.8
Palmitoleic (C16:1)	0.1	0.0	0.1	0.1	0.0	0.6	0.9	0.0	0.0
Stearic (C18:0)	3.6	1.6	6.6	3.7	1.8	1.4	6.9	20.1	0.9
Oleic (C18:1)	24.9	33.0	46.1	25.2	53.3	65.6	41.1	57.9	28.2
Linoleic (C18:2)	53.0	20.4	8.6	63.1	28.4	25.2	34.7	24.7	9.3
Linolenic (C18:3)	6.1	7.9	0.3	0.2	0.3	0.1	0.3	7.9	23.9
C18:4	0.0	0.0	0.0	0.0	0.0	0.0	0.0	0.0	3.7
Arachidic (C20:0)	0.3	0.0	0.4	0.3	0.9	0.1	0.0	0.2	0.0
Eicosenoic (C20:1)	0.3	9.3	0.2	0.2	2.4	0.1	0.2	1.0	0.0
C20:5	0.0	0.0	0.0	0.0	0.0	0.0	0.0	0.0	3.4
Behenic (C22:0)	0.0	0.0	0.1	0.7	3.0	0.0	0.0	0.2	0.0
Erucic (C22:1)	0.3	23.0	0.0	0.1	0.0	0.1	0.0	0.2	0.0
Lignoceric (C24:0)	0.1	0.0	0.1	0.2	1.8	0.1	0.0	0.0	0.0
Nervonic (C24:1)	0.0	0.0	0.0	0.0	0.0	0.0	0.0	0.0	0.0

^1^ Cn:m: n is the number of carbon atoms and m is the number of double bonds.
